# Spatiotemporal Heterogeneity of Chlorophyll Content and Fluorescence Response Within Rice (*Oryza sativa* L.) Canopies Under Different Nitrogen Treatments

**DOI:** 10.3389/fpls.2021.645977

**Published:** 2021-03-25

**Authors:** Jiafei Zhang, Liang Wan, C. Igathinathane, Zhao Zhang, Ya Guo, Dawei Sun, Haiyan Cen

**Affiliations:** ^1^College of Biosystems Engineering and Food Science, State Key Laboratory of Modern Optical Instrumentation, Zhejiang University, Hangzhou, China; ^2^Key Laboratory of Spectroscopy Sensing, Ministry of Agriculture and Rural Affairs, Hangzhou, China; ^3^Key Laboratory of Modern Precision Agriculture System Integration Research, Ministry of Education of China, China Agricultural University, Beijing, China; ^4^Key Laboratory of Agriculture Information Acquisition Technology, Ministry of Agriculture of China, China Agricultural University, Beijing, China; ^5^Department of Agricultural and Biosystems Engineering, North Dakota State University, Fargo, ND, United States; ^6^Key Laboratory of Advanced Process Control for Light Industry, Ministry of Education, Jiangnan University, Wuxi, China

**Keywords:** heterogeneity, phenotyping, chlorophyll content, chlorophyll *a* fluorescence, OJIP transients, photosynthesis, nitrogen

## Abstract

Accurate acquisition of plant phenotypic information has raised long-standing concerns in support of crop breeding programs. Different methods have been developed for high throughput plant phenotyping, while they mainly focused on the canopy level without considering the spatiotemporal heterogeneity at different canopy layers and growth stages. This study aims to phenotype spatiotemporal heterogeneity of chlorophyll (Chl) content and fluorescence response within rice leaves and canopies. Multipoint Chl content and high time-resolved Chl *a* fluorescence (ChlF) transient (OJIP transient) of rice plants were measured at different nitrogen levels and growth stages. Results showed that the Chl content within the upper leaves exhibited an increasing trend from the basal to the top portions but a decreasing pattern within the lower leaves at the most growth stages. Leaf Chl content within the rice canopy was higher in the lower leaves in the vegetative phase, while from the initial heading stage the pattern gradually reversed with the highest Chl content appearing in the upper leaves. Nitrogen supply mainly affects the occurrence time of the reverse vertical pattern. This could be the result of different nutritional demands of leaves transforming from sinks to sources, and it was further confirmed by the fall of the JI phase of OJIP transient in the vegetative phase and the rise in the reproductive phase. We further deduced that the vertical distribution of Chl content could have a defined pattern at a specific growth stage. Furthermore, the reduction of end acceptors at photosystem I (PSI) electron acceptor side per cross section (RE_0_/CS) was found to be a potential sensitive predictor for identifying the vertical heterogeneity of leaf Chl content. These findings provide prior knowledge on the vertical profiles of crop physiological traits, which explore the opportunity to develop more efficient plant phenotyping tools for crop breeding.

## Introduction

Rice (*Oryza sativa* L.) is a critical staple crop that feeds more than half of the world’s population. The increasing population, coupled with shrinking cropland, triggers the demand for improving rice grain yield to address the global food issues (Pandey et al., 2010). In recent decades, approaches that incorporate phenomic and genomic technologies enable high throughput screening of crops with higher productivity, while the acquisition of crop phenotypic data in field trials remains a technical bottleneck (Ghanem et al., 2015; Reynolds and Langridge, 2016; Furbank et al., 2019). Previous research efforts have been devoted to phenotype a variety of key plant traits, such as the contents of chlorophyll (Chl) and other photosynthetic pigments, nitrogen, and also water, on a laboratory scale using optical sensors. However, these studies mainly focused on the canopy level without considering the spatiotemporal heterogeneity within the leaf and/or canopy (Hikosaka, 2016; Li et al., 2019).

The physiological performance of leaves can vary in different positions. For example, leaf nitrogen concentration tended to be higher in photosynthetically active leaves of the upper layer, especially when nitrogen supply is deficient, and could theoretically track the within-canopy light gradient (Hikosaka, 2016; Pao et al., 2018). This vertical heterogeneity within crop canopies could be regarded as an adaptive strategy of continuous adjustment of between- and within-leaf partitioning to maximize canopy photosynthesis rate in response to limited nutritional resources and fluctuating environmental conditions (Adachi et al., 2017; Pao et al., 2018). Meanwhile, effects arising from crop growth and development dynamics might result in divergent optimal vertical distribution strategies. Photoassimilates in mature leaves and other nutrients are translocated to developing leaves (sink) during the vegetative phase, while the developing grains become the growth centers/sinks during the reproductive phase (Yu et al., 2015). Quantitative analysis of the spatiotemporal heterogeneity within the canopy is thus indispensable for accurate phenotyping.

As the primary light-harvesting pigment and the reaction center (RC), Chl directly affects the light interception, penetration, and conversion in the plant, hence the photosynthetic capacity and crop productivity (Evans, 1989; Croft et al., 2017). It was expected that rice plant leaves with reduced Chl content would increase canopy photosynthesis for the individual plant as it permits more light penetration into the lower layers and dissipates less light energy via non-photochemical quenching (NPQ) in the flag leaves (Gu et al., 2017; Walker et al., 2018). Therefore, one promising strategy for improving grain yields is to increase canopy photosynthesis by manipulating the vertical distribution of leaf Chl content (Ort et al., 2011; Yin and Struik, 2015; Gu et al., 2017). Investigations into the vertical distribution of leaf Chl showed different results in several crops. Maize canopies exhibited a bell-shaped curve with the highest Chl content positioned near the intermediate layer leaves during the reproductive phase (Ciganda et al., 2008; Winterhalter et al., 2012; Li et al., 2019). While winter wheat showed an increasing trend from the bottom to top with the highest Chl content located at the flag leaf (Huang et al., 2011). The vertical distribution of leaf Chl content seems to follow the source-sink regulation with the highest concentration in the ear leaf of maize and the flag leaf of winter wheat, both of which serve as the main source of carbohydrates for grain (sink) filling. On the other hand, it has been reported that the vertical gradient of Chl content along the canopy height were partly attributed to acclimation to light penetration (Kull, 2002; Morales and Kaiser, 2020). Leaves with low irradiance tend to have a higher Chl proportion to offset the reduction of electron transport capacity per unit, which was the result of chloroplast movement inside the cell (Kasahara et al., 2002; Kull, 2002; Li et al., 2018). In general, these studies have demonstrated the existence of vertical distribution of leaf Chl content, but the interaction of plant species, growth stages, and prevailing environment must be considered to obtain a quantitative basis for plant heterogeneity.

In addition to Chl content, Chl *a* fluorescence (ChlF) serves as an indicator of plant physiological status (Baker and Rosenqvist, 2004; Ripoll et al., 2016). The energy absorbed by Chl molecules generally competitively undergoes three forms of transformation: (i) photochemistry; (ii) dissipation as heat; and (iii) ChlF (Govindje, 1995; Kalaji et al., 2014; Stirbet et al., 2020). Compared with traditional gas exchange measurements, ChlF techniques have come to be a more effective, non-invasive, and high throughput approach to understand spatiotemporal dynamics of photosynthesis and provides valuable information about leaf light energy absorption, transport, and dissipation (Baker and Rosenqvist, 2004; Papageorgiou and Govindjee, 2004; Baker, 2008). To date, ChlF and various calculated parameters, such as *F_v_/F_m_* (maximum photosystem II quantum yield) (Papageorgiou, 2007; Stirbet et al., 2020), have been intensively adopted to diagnose the physiological status of plants exposed to abiotic stress conditions (e.g., chilling and high temperature, salinity, drought, heavy metals, and nutrient deficiency) (Van Kooten and Snel, 1990; Ač et al., 2015; Kalaji et al., 2016; Mishra et al., 2019). Several studies have recognized that significant spatial differences of photosystem II (PSII) activity and energy usage efficiency can be observed in different vertical layers via using ChlF parameters (Meng et al., 2001; Feng et al., 2015; Larbi et al., 2015). Feng et al. (2015) have pointed out that the spatial difference of ChlF parameters between the first-second and the firth leaves in the upper layer of wheat could indicate its nitrogen status. Therefore, detecting the ChlF responses offers an alternative method to phenotype spatiotemporal variations of leaf physiological traits.

Given the present research background, this study was to explore the spatiotemporal heterogeneity of leaf Chl content and fluorescence response within rice leaves and canopies. Focusing on rice plants, the specific goals were to: (1) investigate the dynamic vertical profiles of leaf Chl content and fast OJIP transients under different nitrogen treatments; (2) analyze the associations between Chl content and ChlF parameters; and (3) extract the inherent characteristics of plant physiological activities and the underlying response mechanism to variations during the vegetative and reproductive phases.

## Materials and Methods

### Plant Materials and Growth Conditions

Rice hybrid cultivar “Yongyou 1540” was grown in the experimental station at Grain-production Functional Area of Anhua, Zhuji City, Zhejiang Province in China (29°31′5.35′′N, 120°6′6.12′′E) in the growing season of 2018 with the average temperature of 29.5°C and total precipitation of 674 mm. Three nitrogen rates, namely 0 (N0), 240 (N1), and 480 (N2) kg ha^–1^ were tested in this experiment. Using a randomized complete block design with four replications, an experimental field consisted of 12 plots with individual plot sized 8 × 5 m was planted. The nitrogen fertilizer was applied three times with the amount of 40, 30, and 30% at the pre-planting, tillering, and booting stages, respectively. The phosphorus (P) and potassium (K) fertilizers were applied in the form of Ca (H_2_PO_4_)_2_H_2_O and KCl with the amount of 120 and 240 kg ha^–1^, respectively, for all the treatments before transplanting. Normal field management, such as irrigation, weed control, was practiced for minimizing the effect of other factors influencing the results.

**FIGURE 1:**
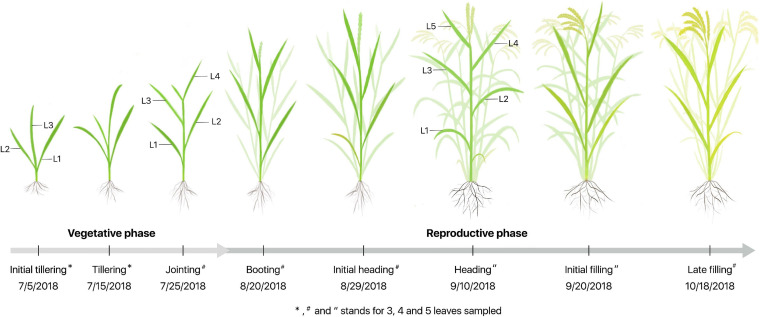
Schematic diagram for sampling and measurements from the initial tillering to the late filling stages during vegetative and reproductive phases. L1–L5 represent the leaf position from bottom to top of the plant, and V1–V3 and R1-R5 are related to eight growth stages, respectively.

The experiment was performed at eight growth stages that were further divided into vegetative (V) and reproductive (R) phases denoted as V1-V3 and R1-R5 in Figure 1, respectively. Four plants in each plot were randomly selected, and all the measurements were performed on three to five fully expanded leaves labeled as L1, L2, L3, L4, and L5 from the bottom to top of each plant, depending on the growth stages.

### Measurements of Chlorophyll Fluorescence *in vivo*

Measurements of fast ChlF transient of the attached rice leaves were performed with a continuous excitation chlorophyll fluorimeter (Handy PEA, Hansatech Instruments Ltd., Norfolk, United Kingdom) in the field from 11:00 am to 1:00 pm. Leaf samples were first dark adapted by using a leaf clip with the measurement area of 4 mm diameters in the middle of the adaxial side of each fully expanded leaf but avoiding the midrib and edges. After dark adaptation for 20 min (Bolhar-Nordenkampf et al., 1989), all the reaction centers (RCs) are fully oxidized and are available for photochemistry. The dark-adapted leaf was then immediately illuminated with a saturating light emitted by an array of three ultra-bright red LED at 650 nm with an intensity of 3,000 μmol m^–2^ s^–1^. A rapid polyphasic curve was then obtained with a fast rise of approximately 1 s, followed by a slow decline of approximately 120 s to the steady-state *F*_s_ of ChlF, which is known as Kautsky fluorescence induction (Kautsky and Hirsch, 1931).

### Measurements of Chlorophyll Content

After the ChlF measurement in the field, the labeled plants were cut, placed in the sealed bags, and stored in a cooler with a temperature of about 2°C. All samples were immediately transported to the laboratory (Zhejiang University, Zhejiang Province, China) for further analysis. Leaf disks with a diameter of 0.85 cm were collected from the basal, central, and top portions of the leaf. Each leaf disk was put in a tube with 1.8 mL of 95% ethanol, and soaked in the dark environment for 24 h. A spectrophotometer (Epoch, BioTek Instruments, Winooski, United States) was then used to measure the absorbance of light at 470, 649, and 665 nm, and the Chl content (μg cm^–2^) was finally calculated using a standard curve (Lichtenthaler and Wellburn, 1983).

### Description of OJIP Transient and JIP Test

The fast fluorescence rise (O-J-I-P) curve usually exhibits the step J (at 2 ms) and I (at 30 ms) between the initial O (*F*_0_) and the maximal fluorescence P (*F*_P_); and the data were recorded every 10 μs for the first 2 ms and every 1 ms thereafter (Strasser, 1992). As shown in Supplementary Figure 1, the OJ phase is the photochemical process related to the reduction of primary quinone electron acceptors (Q_A_) to Q_A_^–^. A majority of Q_A_^–^ that cannot be timely oxidized by second quinone electron acceptors (Q_B_) is accumulated rapidly, causing the fluorescence intensity to increase instantaneously because it only takes ∼250 ps for the electron transferring from pheo^–^ to Q_A_, but 0.1∼0.6 ms from Q_A_ to Q_B_ (Govindjee and Björn, 2017). The intermediate phase JI that corresponds to the reduction of plastoquinone (PQ) pool needs much more time, approximately 1∼20 ms (Govindjee and Björn, 2017), due to the physical distance between PSII and cytochrome *b_6_f* (Cyt *b_6_f*) complex. The IP phase parallels the reduction of acceptors in and around photosystem I (PSI), namely PC^+^ and P700^+^ (Schansker et al., 2003, 2005).

The JIP test provides in-depth analytical information behind the shape change of OJIP transients, with computing a series of phenomenological and biophysical parameters to quantify the PSII behavior (Tsimilli-Michael et al., 2000; Strasser et al., 2004). It is defined as the relative variable fluorescence at time *t*, *V*_t_, which is the ratio of the variable fluorescence to the maximal variable fluorescence and is expressed as

(1) $$V_{t}=\left(F_{t}-F_{0}\right)/\left(F_{M}-F_{0}\right)$$

where *F*_t_ is the variable fluorescence at time *t*, *F*_0_ is the initial fluorescence at t = 0 (or 50 μs), and *F*_M_ is the maximum fluorescence. Thus, the relative variable fluorescence at J-step (*V*_J_) in terms of fluorescence at J-step (*F*_J_) is calculated as

(2) $$V_{J}=\left(F_{J}-F_{0}\right)/\left(F_{M}-F_{0}\right)$$

*M*_0_ is the approximated initial slope of the relative variable fluorescence, with reference to the fluorescence at 300 μs (*F*_300μ*s*_), is described as

$$M_{0}={\left(△\,V/△\,t\right)}_{0}=\left(V_{300\,\mu \,s}-V_{50\,\mu \,s}\right)/\left(0.25\,m\,s\right)$$

(3) $$=4\,\left(F_{300\,\mu \,s}-F_{0}\right)/\left(F_{M}-F_{0}\right)$$

Then, the calculated functional parameters, including the specific (per RC) and phenomenological (per excited cross-section, CS) energy fluxes for absorption, trapping, electron transport, and dissipation, can be obtained. Other parameters, such as quantum yields, the density of reaction centers, and performance index were also derived. Detailed descriptions of these parameters used in this study are summarized in Table 1 (Strasser et al., 2004).

**TABLE 1:** Formulae and glossary of terms used in the JIP-test analysis.

**Formulae and terms**	**Illustrations**
**Original parameters extracted from the fluorescence transient O-J-I-P**	
*F*_t_	Fluorescence intensity at time *t*
*F*_*J*_ ≡ *F*_2ms_	Fluorescence intensity at the J-step (2 ms) of the OJIP transient
*F*_*I*_ ≡ *F*_30ms_	Fluorescence intensity at the I-step (30 ms) of the OJIP transient
*F*_P_	Maximum fluorescence intensity at the peak of the OJIP transient
*t*_F_M_	Time required to reach the maximum fluorescence after dark adaptation
*Area*	Total complementary area between fluorescence induction and *F = F*_M_

**Fluorescence parameters derived from original parameters**	
*F*_0_	Minimum Chl *a* fluorescence yield in the dark-adapted state
*F*_M_ = *F*_P_	Maximum Chl *a* fluorescence yield in the dark-adapted state
*F*_v_ = *F*_t_ – *F*_0_	Variable fluorescence at time *t*
*F*_V_ = *F*_M_ – *F*_0_	Maximum variable fluorescence
*S*_m_ ≡ *Area*/(*F*_M_ – *F*_0_)	The normalized total complementary area above the OJIP transient
*N*	The number of Q_A_ reduction from time 0 to *t*_*F_M*_

**Specific energy fluxes [per Q_A_^–^ reducing PSII reaction center (RC)]**	
*ABS/RC = M*_0_⋅ (1/*V*_*J*_)⋅ (1/*φ*_Po_)	Absorption flux per RC
*TR_0_/RC = M*_0_⋅ (1/*V*_*J*_)	Trapped energy flux per RC (at *t* = 0)
*ET_0_/RC = M*_0_⋅ (1/*V*_*J*_)⋅*ψ*_Eo_	Electron transport flux per RC (at *t* = 0)
*DI_0_/RC* = (*ABS*/*RC*) – (*TR_0_/RC*)	Dissipated energy flux per RC (at *t* = 0)
*RE_0_/RC = M*_0_⋅ (1/*V*_*J*_)⋅*ψ*_Eo_⋅*φ*_Ro_	Reduction of end acceptors at PSI electron acceptor side per RC (at *t* = 0)

**Quantum yields or flux ratios**	
*φ*_Po_ ( = *F_v_/F_m_*) ≡ *TR_0_/RC* = [1 – (*F*_0_*/F*_M_)]	The maximum quantum yield for primary photochemistry (at *t* = 0)
*ψ*_Eo_ ≡ *ET*_0_*/TR*_0_ = (1–*V*_*J*_)	The probability that a trapped exciton moves an electron into the electron chain beyond Q_A_^–^ (at *t* = 0)
*φ*_Eo_ ≡ *ET*_0_*/ABS* = [1 – (*F*_0_*/F*_M_)]⋅(1–*V*_*J*_)	Quantum yield for electron transport (at *t* = 0)
*φ*_Ro_ ≡ *RE*_0_*/ABS* = [1 – (*F*_0_*/F*_M_)]⋅(1–*V*_*I*_)	Quantum yield for the reduction of the end acceptors of PSI per photon absorbed

**Phenomenological energy fluxes [per excited cross-section (CS)]**	
*ABS/CS ≈ F*_M_	Absorption flux per CS
*TR*_0_*/CS = φ*_Po_⋅ (*ABS*/*CS*)	Trapped energy flux per CS (at *t* = 0)
*ET*_0_*/CS = φ*_Po_⋅*φ*_Eo_⋅ (*ABS*/*CS*)	Electron transport flux per CS (at *t* = 0)
*DI*_0_*/CS* = (*ABS*/*CS*) – (*TR_0_/CS*)	Dissipated energy flux per CS (at *t* = 0)
*RE*_0_*/CS = φ*_Eo_⋅*φ*_Ro_⋅ (*ABS*/*CS*)	Reduction of end acceptors at PSI electron acceptor side per CS (at *t* = 0)

**Density of reaction centers**	
*RC/CS = φ_Po_* – *(V*_*J*_*/M*_0_)⋅ *(ABS/CS)*	Density of RCs (Q_A_-reducing PSII centers)

**Performance indexes**	
*PI*_ABS_ = $\left(\frac{R\,C}{A\,B\,S}\right)\,\left(\frac{\varphi_{\text{P\_o}}}{1-\varphi_{\text{P\_o}}}\right)\,\left(\frac{\psi_{o}}{1-\psi_{o}}\right)$	Performance index on absorption basis

### Statistical Analysis

Statistical analysis was performed using SPSS 14.0 software (SPSS Inc., Chicago, IL, United States). One-way analysis of variance (ANOVA) with Fisher’s least significant difference (LSD) test was used to evaluate the significance of different levels on measured parameters. The standard error (SE) of replicates at each nitrogen level was calculated from the standard deviation (*SD*) and the number of replicates (*n*) as SE = (*SD*/n^0.5^). In this study, four replications were used.

To further establish the linkage between the Chl content and the photosynthetic activities, we used an eXtreme Gradient Boosting (XGBoost) regression method (Chen and Guestrin, 2016), which defines the most important features and outperforms in solving scale problems with a minimal amount of resources. The mechanism of XGBoost is to keep constructing and training a new tree which takes the features as nodes and the corresponding instance scores as leaves. Specifically, a new tree is created to fit residual errors of last iteration. The objective function of XGBoost consisting of a loss function and a regularization function, can be expressed as:

(4) Obj=(t)∑li=1n(yi,y^i(t-1)+ft(xi))+Ω(ft)

where *l* is a differentiable convex loss function that measures the difference between the prediction ${\hat{y}}_{i}$ and the target *y_i*, and y^i(t-1) denotes the prediction of the i-th instance at the t-th iteration. *f*_*t*_(*x*_*i*_) is the outcome of input *x_i* for the t-th tree. The regular termΩ measures the complexity of the training model to avoid over-fitting. Here *n* is the quantity of trees.

In this study, we constructed a data set with Chl content as the dependent variable and JIP parameters as the independent variable for each leaf layer to evaluate the differential contributions of JIP parameters to Chl content variations. The XGBoost modeling used 80% data for training and the remaining 20% for testing. To quantify the model performance, the root mean square error (RMSE) that determines the optimal training parameters of maximal tree depth, minimal children weight, and learning rate in 5-fold cross-validation, which is defined as

RMSE=1n∑(yi-y^i)21n

where *y_i* and ${\hat{y}}_{i}$ represent the measured and the predicted values, respectively, and *n* is the sample number. And the GridsearchCV function was applied to find the optimal parameters. The number of boosted trees (*n_estimators*) of our model was set to 100. The model development and parameter optimization were performed in Python (version 3.5; Python Software Foundation, Wilmington, Delaware, United States), using the xgboost package.

## Results

### Spatiotemporal Variations in Chlorophyll Content Within Rice Leaves and Canopies

**FIGURE 2:**
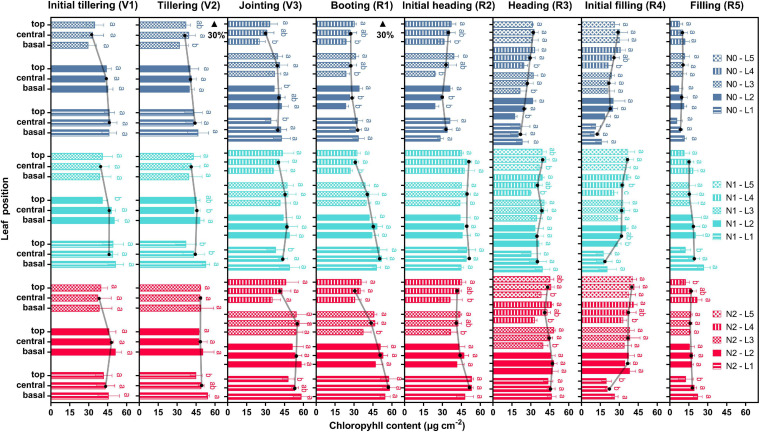
Dynamics of leaf Chl content during different growth stages. The histogram shows the variations of the Chl content within a single leaf and the vertical distribution of the canopy (L1–L5). The solid lines with black dots are averages of basal, central, and top values. Error bars indicate the standard deviation (SD) of four replicates. Values within a leaf followed by the same letter are not significantly different (*P* ≥ 0.05, Fisher’s LSD test). Black arrows denote the timing of topdressing of nitrogen fertilizer.

The variations of Chl content at the leaf level and its vertical distribution at the canopy level under different nitrogen treatments are shown in Figure 2 and Supplementary Table 1. Leaf Chl content increased with higher nitrogen fertilizer and was saturated when nitrogen fertilizer excessive. No obvious change of the average Chl content was observed from the initial tillering (V1) to the jointing (V3) with the treatments of N0 and N1, while there was a considerable increase for plants under the N2 treatment. From the jointing (V3) to the booting (R1), the average Chl content reduced by 21.9, 5.6, and 10.7% in the N0, N1, and N2 treatments, respectively. The 11.7 and 11.6% increase of Chl content were further observed in the N0 and N1 treatments, respectively, from the booting (R1) to the initial heading (R2), but it slightly decreased in the N2 treatment. From then on, the average Chl content of plants under all treatments decreased significantly until the late filling (R5). Notably, the topdressing at the tillering (V2) may result in the subsequent increase of Chl content from the tillering (V2) to the jointing (V3) in N1 and N2 groups, however, another topdressing at the booting (R1) only seems to work on N1 group, but not on the N2.

The Chl content showed a significant spatiotemporal heterogeneity within rice canopies, with similar changing patterns in all the three treatments from the base of the leaf blade to the top, from the bottom to top layers of the canopy, and from the initial tillering (V1) to the late filling (R5) stages. For the N1 treatment, the leaf Chl content of the upper layers (L3, L4, and L5) exhibited an increasing tendency from the basal to the top portions. Leaves in the lower layers (L1 and L2) generally presented a decreasing pattern, except the upper leaves at the late filling (R5) and the lower leaves at the booting (R1) and initial heading (R2). This suggested that it might occur in an acropetal direction of photosynthate translocation in the upper leaves whereas in a basipetal direction of that in the lower leaves. Moreover, the highest Chl content can be observed in the lower leaves from the initial tillering (V1) to the booting (R1), while a reversed pattern was observed starting from the initial heading (R2). It finally showed a relatively uniform distribution of Chl content in a vertical profile at the late filling (R5). Similar patterns were found in plants with the N0 and N2 treatments, but the turning point appeared earlier for N0 and later for N2, which indicated a shortened and prolonged vegetative phase, respectively. Additionally, the leaf Chl content of each leaf position in plants with the N0 treatment significantly decreased (*P* < 0.05) from the jointing (V3) to the booting (R1) but only the upper leaves decreased in the N1 and N2 treatments, hence a steeper Chl gradient of them at the booting (R1) was observed compared with that in N0.

### Characterization of Chlorophyll Fluorescence Response With OJIP Transient Curves

**FIGURE 3:**
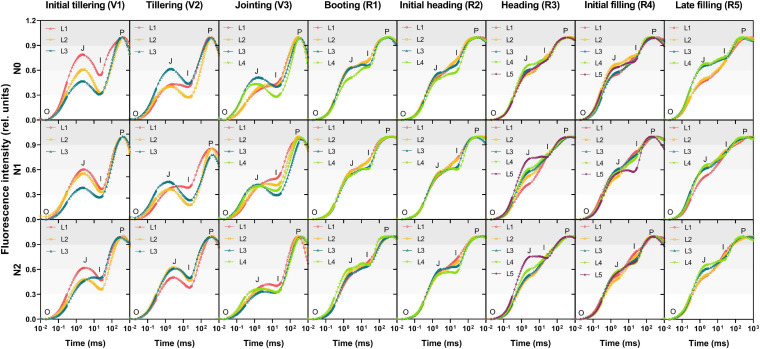
The OJIP transients in leaves along the vertical distribution of the canopy during different growth stages. Curves are presented in the logarithmic time scale with the normalization by F_0_ and F_P_ to the P-level for eliminating the difference in light intensity. O (origin, all RCs open) is the minimum fluorescence level, P (peak, all RCs closed) is the maximum level, and J (∼2 ms) and I (∼30 ms) are intermediate inflections. Individual data points are the mean value of four biological replicates. L1–L5 represent the leaf position from bottom to top of the plant.

To investigate the spatial and temporal variations of the ChlF response of different leaves within the canopy, OJIP transient curves under three nitrogen treatments were determined as shown in Figure 3. During the reproductive phase, transients of the leaves with the N0, N1, and N2 treatments all showed a typical polyphasic rise O-J-I-P shape, while it presented an evident JI-fall with a gradually decreased amplitude during the vegetative phase, indicating an inhomogeneity of photochemistry performance within leaves at different growth stages. There was no significant difference in OJIP transients between vertical layer leaves at the booting (R1) and the initial heading (R2) while it showed obvious vertical heterogeneity at other stages: initial tillering (V1) and late filling (R5). The intensity of the J step in the upper leaves was higher than that in the lower at the initial tillering (V1) and the opposite occurred at the late filling (R5). It should be noted that the transients of the L1 and L2 layer leave with the N0 treatment and the L1 layer leaves with the N1 treatment initially showed the normal JI-rise characteristic at the jointing (V3), when all the leaves with the N2 treatment had not transformed yet. It further demonstrated the difference of different nitrogen treatments in plant growth rate as discussed above. However, the vertical difference in the OJIP transients among the three nitrogen levels appeared two stages earlier than that in the distribution of Chl content.

### Characterization of Leaf Physiological Activities With JIP Parameters

The representative JIP parameters were further selected to study the heterogeneity of physiological activities in the vertical layer leaves. These functional parameters varied in different vertical layer leaves and growth stages, thus presenting various spatiotemporal patterns. The changing patterns were generally the same among N0, N1, and N2 treatments at each growth stage (Supplementary Figure 2). Note that the vertical pattern of N2 at the tillering (V2) and N0 at the initial heading (R2) differed from the other two treatments. At the tillering (V2), the JIP parameters (Table 1) of *S*_m_, *N*, *ABS/RC*, *DI_0_/RC*, *TR_0_/RC*, and *RE_0_/RC* of the upper leaves with the N2 treatment were smaller than that of the lower leaves, indicating a growth lag of the upper leaves with surplus nitrogen fertilizer. Likewise, the vertical pattern of the initial heading (R2) seemed to be transformed in advance since it tended to present the same pattern with the next late filling (R5). *RE_0_/RC*, *RE_0_/CS*, and *φ_Ro_* of the upper leaves at this stage were not superior to the lower leaves, which were the prominent characteristics in plants under the N1 and N2 treatments.

**FIGURE 4:**
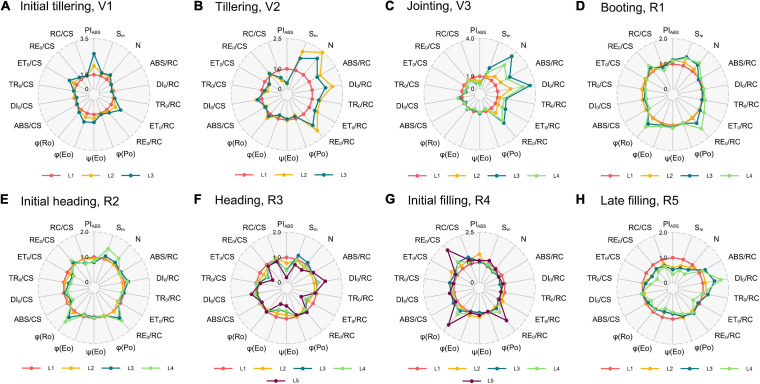
Radar plots of the N1 treatment with a series of important parameters derived from experimental fast OJIP transients during different growth stages [**(A)**, initial tillering; **(B)**, tillering; **(C)**, jointing; **(D)**, booting; **(E)**, initial heading; **(F)**, heading; **(G)**, initial filling; **(H)**, late filling]. These parameters for each leaf are the average of all samples with the N1 treatment. Taking each leaf at the L1 position as the control (each parameter equals 1, denoted by a red circle) and parameters of another leaf of the stage are expressed by fraction relative to the corresponding value of L1 layer leaf. Individual data points are the mean value of four biological replicates. L1–L5 represent the leaf position from bottom to top of the plant.

Considering the results of the N1 treatment as a representative (Figure 4), parameters of the % growth stages can be divided into four categories, with dissimilar peaks and valleys that indicated an increasing and decreasing tendency from the bottom to top, respectively. The first category was shown at the initial tillering (V1, Figure 4A), with a prominent peak in *PI*_ABS_, *ET_0_/RC*, *ET_0_/CS*, *φ_Eo_*, and *ψ_Eo_*. This pattern suggests that the upper leaves might possess a relatively high electron transport efficiency between PSII and PSI than the lower leaves. There existed no identical peaks at the tillering (V2, Figure 4B) and jointing (V3, Figure 4C) but *N*, *ABS/RC*, *TR_0_/RC*, *DI_0_/RC*, and *RE_0_/RC* strongly increased in the upper leaves, concomitant with a valley in active PSII RCs (*RC/CS*) and *PI*_ABS_. Hence, we classified the tillering (V2) and jointing (V3) into the second category. This indicated that the upper leaves at these growth stages absorbed more energy, but the amount of active PSII RCs was relatively low, which in turn increased the absorption, dissipation, and electron transport per RC, and decreased the performance index *PI*_ABS_. For the third category in the booting (R1, Figure 4D) and initial heading (R2, Figure 4E), it shared the same peak with the second in *RE_0_/RC*, and there were new peaks in *RE_0_/CS* and *φ_Ro_*. Surprisingly, similar results were observed in the initial filling (R4, Figure 4G) rather than the coming heading stage (R3, Figure 4F). The most striking characteristics of this pattern were that the PSI electron transport efficiency was higher in the upper leaves as stepping into the reproductive phase. The last category with two peaks in *DI_0_/RC* and *DI_0_/CS*, and four distinct valleys in *PI*_ABS_, *ET_0_/RC*, *φ_Eo_*, and *ET_0_/CS* was of the heading (R3, Figure 4F) and late filling (R5, Figure 4H), where the dissipated energy was more significant and the electron transport between PSII and PSI became less efficient in the upper leaves in contrast to that in the initial tillering (V1). Interestingly, the results of the four critical growth stages (e.g., tillering—V2, jointing—V3, initial heading—R2, and late filling—R5) were almost the same as our repeated experiments in 2019 (Supplementary Figure 3).

**FIGURE 5:**
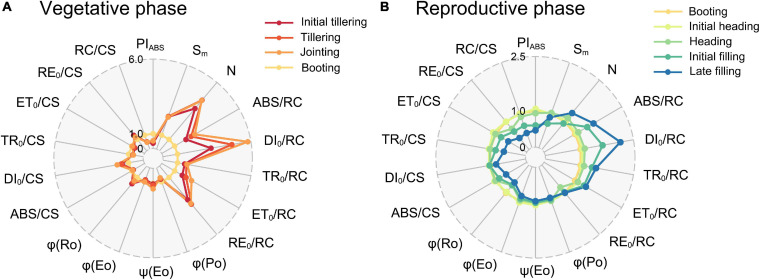
Radar plots of functional parameters in the **(A)** vegetative and **(B)** reproductive phases. The mean value of all parameters at the booting (R1) was considered as the control (each parameter equals 1, denoted by the red circle), and parameters of leaves at the other stages were expressed by the fraction of the average value at the booting (R1). Individual data points are the mean value of four biological replicates.

Comparison of these functional parameters of different growth stages illustrated in Figure 5 provides information about the temporal variation of photosynthetic capacity. It can be observed that *N*, *ABS/RC*, *DI_0_/RC*, *ET_0_/RC*, and *RE_0_/RC* of the vegetative phase (Figure 5A) were mostly more than twice that of the booting (R1). Furthermore, the ratio of *DI_0_/RC* between the jointing (V3) and the booting (R1) was even close to 6.0. However, they all showed an increasing trend from the initial tillering (V2) to the jointing (V3) but decreased until the booting (R1). The S_m_ was constant during the vegetative phase, but it decreased by 23% at the booting (R1). *ABS/RC*, *DI_0_/RC*, *TR_0_/RC*, and *ET_0_/RC* increased during the reproductive phase (Figure 5B), and thus they achieved a minimum value at the booting (R1). By contrast, *PI*_ABS_ and *RC/CS* initially increased and then decreased after the booting (R1), thereby reaching a maximum value at the booting. In addition, PSI activity-related parameters (*RE_0_/CS* and *φ_Ro_*) kept decreasing throughout the whole growth period. Interestingly, the comparison result between the stages in the vegetative phase (Figure 5A), as well as between the stages in the reproductive phase (Figure 5B) was similar to the vertical pattern at the jointing (V3, Figure 4C) and the late filling (R5, Figure 4H), respectively.

### Linkages Between Leaf Chl Content and JIP Parameters

**FIGURE 6:**
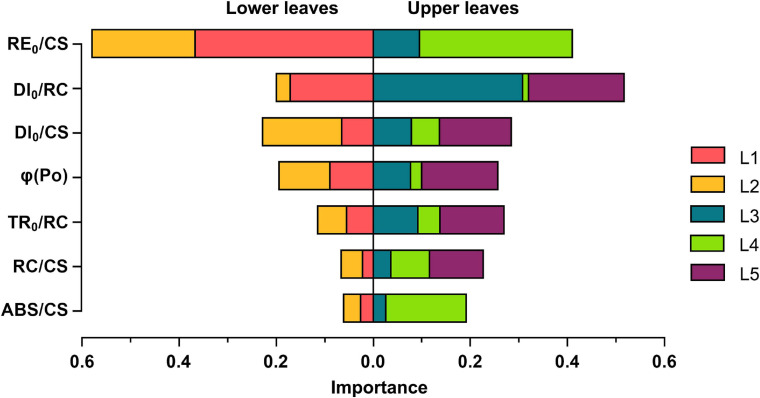
Contribution of the JIP parameters to the Chl content estimation of each vertical leaf layer based on the XGboost tree regression model and the cumulative feature importance of the lower leaves vs. the upper leaves. L1-L5 represent the leaf position from bottom to top of the plant.

To reveal the linkages between leaf photosynthetic activities and Chl content, the XGboost algorithm was applied to determine the ranking of JIP parameters, and the top seven important parameters were fed into the model (Figure 6). *RE_0_/CS* and *DI_0_/RC* have the top two weights, indicating that the Chl content may have significant effects on the electron transport capacity within PSI per CS and the energy dissipation per RC. The importance of them varied across leaf layers but shared certain characteristics: *RE_0_/CS* was of more importance to correlate with Chl content in L1, L2, and L4 leaf layers while *DI_0_/RC* was the predominant variable in both L3 and L5. Notably, *DI_0_/RC* exhibited a stronger connection with Chl content in the upper leaves, particularly in L3, than the lower leaves, and *ABS/CS* in L4 leaf layer obtained high importance scores.

## Discussion

### Vertical Distribution of Chlorophyll Content and the Effects of Nitrogen Fertilizer

Significant differences exist among different nitrogen-supplied rice plants concerning their Chl contents and vertical distributions. Increasing the nitrogen supply does increase the overall Chl content of plants to a certain extent, but this effect on the vertical leaf layer varies in different growth stages. We demonstrated that the Chl content within the rice canopy exhibits different vertical patterns at specific growth stages. The Chl content tends to be higher in the lower leaves during the vegetative phase, while from the initial heading, the pattern is gradually reversed with the highest Chl content positioned in the upper leaves.

The acquisition and utilization of nitrogen for the vegetative growth of young developing leaves mainly depend on the uptake, assimilation, and transport of the soil nitrogen (Tegeder and Masclauxdaubresse, 2018). Thus, both the pre-planting nitrogen supply and the topdressing played a pronounced role in increasing the Chl content of each layer, preferentially the lower leaves, from the initial tillering to the jointing (V1–V3, Figure 2). It gradually appeared a vertical gradient that the leaf Chl content increased from the top to bottom. While entering the reproductive phase, the mature leaves, especially the flag leaves, become the most timely and efficient source of sucrose for grain (sink) filling (Li et al., 2013; Yu et al., 2015). At this time, the Chl content of the upper leaves all decreased considerably in different nitrogen-supplied rice plants, thereby producing a more remarkable vertical Chl gradient at the booting (R1). However, the gradient of plants with the N0 treatment was not as steep as that with the N1 and N2 treatments. This is likely due to that the nutrients provided by the upper leaves of N0 plants cannot fully meet the demands and their lower leaves transported nutrients up to the developing grains as well. With the grain develops (heading stage, R3), nitrogen utilization for grain would rely more on the pre-stored nitrogen in the soil and they were preferentially portioned to the upper layer (Masclaux-Daubresse et al., 2010; Chen et al., 2020), making the Chl content of the uppermost leaves increased from the booting (R1) to the initial heading (R2) even without topdressing. Meanwhile, with various nitrogen in senescing leaves remobilized to develop grains, the Chl content of the lower leaves gradually decreased, a reversed vertical Chl gradient with the highest Chl content in the upper leaves hence formed. When rice plants completed grain filling, nitrogen accessible from the upper leaves may also be depleted (Liu et al., 2018), thus all presented a uniform vertical distribution.

Previous studies have suggested that nitrogen deficiency generally occurs in the lower leaves and triggers the leaf senescence, while excessive nitrogen would primarily influence the upper leaves (Gan and Amasino, 1997; Wang et al., 2005; Huang et al., 2014). However, in our results, the impact of nitrogen supply on the vertical distribution primarily lies in the turning point of its pattern reversed via affecting the duration of the vegetative and reproductive phase. The Chl content of the lower leaves with the N0 treatment indeed reduced a lot compared to the N1 and N2 at the booting (R1), but it did not happen at other growth stages. The vegetative phase of N0 was shortened in contrast to N1, with an early appearance of the reversed vertical distribution pattern. Moreover, the nitrogen fertilizer of the N2 treatment might not be excessive for rice plants in our experiments that no significant difference in the upper leaves was observed. Nevertheless, it still resulted in a prolonged vegetative growth to some extent, delaying the onset of the pattern reversed.

Leaves of different layer play a different role (sources or sinks) in plant growth and development, leading to different nutrient demands which determine the direction of nutrient flow and metabolic pathways (Yu et al., 2015; Paul et al., 2020). We further hypothesize that the different nutritional demands of leaves transforming from sinks to sources might be one of the dominant driving forces causing the spatiotemporal heterogeneity of Chl content. It could have a defined vertical distribution of Chl content at a specific growth stage when there is no intense external stress. While insufficient or excessive nitrogen stress would accelerate or delay the function transformation in rice leaves, respectively, thus affecting the vertical pattern. Cultivar differences would impact the absolute value of leaf Chl content without affecting the change tendency at different growth stages (Peng et al., 2008; Wang et al., 2015; Zhang et al., 2019). Coupled with the source-sink regulation, it can be inferred that the spatiotemporal varations of the Chl content within canopy might be similar in ordinary rice varieties.

### Disparity in the Photosynthesis Electron Transport Chain

For the typical OJIP curve, the OJ-rise is usually attributed to the momentary maximum accumulation of Q_A_^–^ that cannot be timely oxidized by Q_B_. Relatively more time is required for the subsequent filling up of the PQ pool, causing a transient block, which in turn leads to a JI-rise (Strasser et al., 2000, 2004). While in the present study, it showed unusual OJIP transient curves with the intensity of I step considerably below the J step in the vegetative growth leaves (Figure 3). This suggests a much efficient electron transport from PSII to PSI compared to that from Q_A_ to Q_B_ at the PSII acceptor side, including a faster reduction of PQ pools that make an exchange of the reduced Q_B_ molecules for an oxidized PQ molecule and a rapid electron transfer to PSI via the re-oxidation of PQH_2_. As a result, the congestion of electrons during the reduction of PQ pools was less severe and the fluorescence intensity was subsequently decreased at I step. This is likely coupled to the larger PQ size or shorter physical distance among PSII, Cyt *b_6_f*, and PSI in the vegetative leaves, which is the “utilization sinks” that imports carbohydrates and nutrients for highly active metabolisms (Yu et al., 2015). Whereas during the reproductive phase, all the mature leaves serve as a source for grain filling via nitrogen remobilization, often accompanied by various protein degradation. For example, if the D1 protein (bounded to Q_B_) is damaged, Q_A_ to Q_B_ electron transfer is often blocked (Murata et al., 2007). Therefore, as entered the booting stage, the number of Q_A_ (*N*) has been reduced and concomitantly, the electrons from Q_A_^–^ transferred into the electron transport chain (*S*_m_) decreased sharply. Both the J and I steps were significantly elevated, suggesting that the electron transport beyond Q_A_ and PQH_2_ were restricted. Moreover, it can be observed from the difference in the JI-phase at the jointing stage among the three nitrogen treatments, the upper leaves that took the lead in transforming from a sink to source also occurred in a JI-fall previously. Hence, it can be inferred that the fall and rise of the JI phase might be a good indicator to identify the leaf function as either sink or source organs.

### Prominent Spatiotemporal Characteristics of Leaf Photochemistry

Mature leaves would have a larger amount of active PSII RCs and higher photosynthesis performance (*RC/CS* and *PI*_ABS_) than younger and senescing leaves, and the maximum of them can be reached at the heading (R3). At each growth stage, we showed that the density of active PSII RCs (*RC/CS*) increased along the canopy depth, and temporally, it increased with growing and ripening but decreased with senescence. The performance index *PI*_ABS_ (incorporating the parameters γ_RC_, φ_Po_, and *φ_Eo_*), which expressed the overall photosynthetic activity of PSII (Strasser et al., 2004), exhibited a better performance when the rice plant was heading. Besides, the probability electron transferred to PSI from PQH_2_ (RE_0_/RC), and the quantum yield for the reduction of end acceptors of PSI (φ_Ro_) all exhibited the highest value in the youngest leaf and kept decreasing as it grew, suggesting that electron transport efficiency over PSI tends to decline with aging.

Absorbed and dissipated energy in an active RC help to describe the vertical light climate inside the canopy. At each growth stage, leaf *ABS/RC* and *DI_0_/RC* level decreased with the canopy depth, and they reached all the lowest values at the booting stage with a dynamic unimodal variation. The *ABS/RC* represents the total number of photons absorbed by Chl molecules of all active RCs. It is partly influenced by the ratio of active/inactive RCs. When the number of inactive centers increased, the ratio *ABS/RC* increased as well. The leaves of the upper canopy contain less active RCs, thus their *ABS/RC* value would be maximum if each layer absorbs the equivalent energy. However, the energy absorbed by leaves at each vertical layer is not equal, and it often decreases with the canopy depth increases (Hirose and Werger, 1987). This is due to the shading of the upper layer that less light penetrates the lower layers, causing the inhomogeneous distribution of light within the canopy. Therefore, the vertical variation of *ABS/RC* is, arguably, in accordance with the light distribution inside canopies. However, it has to be mentioned that the light distribution inside the canopy could vary in different growth stages. In the vegetative phase, light penetration could decrease due to the dense canopy, while increase with the reduced leaf area at the initial heading stage (Hirose et al., 1989). Likewise, it also explained the dynamic pattern of the ratio of total dissipation to the amount of active RCs (*DI_0_/RC*) in the vertical distribution of canopies at different stages. This may have resulted from that the flag leaf would excessively absorb incoming sunlight, dissipate most of the light energy, and prevent light into deeper leaves (Gu et al., 2017). However, leaves in the lower layers make the best use of the light that reaching the bottom, thus lowering heat dissipation.

### Relationships Between Leaf Chlorophyll Content and Fluorescence Response

The significant difference in vertical heterogeneity of Chl content and OJIP transients under three nitrogen treatments in our studies both existed at a specific stage, but it appeared two stages earlier in the OJIP transients than in the Chl content. Leaf photosynthetic apparatus and its activities, therefore, are more susceptible to nitrogen fertilizer than the Chl content, which may not change rapidly with light intensity and nitrogen stress (Yin and Struik, 2015). This indicated that the ChlF response especially OJIP transients has a high potential to phenotype the nitrogen stress during the vegetative phase, which is of great importance to site-specific N management in rice plants.

Our data also demonstrated that the PSI electron transport efficiency-related JIP parameters *RE_0_/CS* was the most relevant variable to Chl content. The spatiotemporal variations of Chl content may significantly affect the PSI electron transport efficiency, hence it can serve as a potential sensitive predictor of identifying the vertical heterogeneity of leaf physiological status. The importance of *DI_0_/RC* in L3 and *ABS/RC* in L4 suggested that the Chl content of L3 layer leaves are closely related to heat dissipation per RC and L4 layer leaf Chl plays a pivotal role in energy absorption, thus they indicated potential aspects to improve energy utilization efficiency via manipulating the vertical distribution of Chl content. From these findings, we can reliably identify the application of the ChlF response as an effective approach for phenotyping the spatiotemporal physiological status.

## Conclusion

In this study, we investigated the vertical distribution characteristics of leaf Chl content and fluorescence responses within rice canopies at different growth stages as well as the relationship between leaf Chl content and fluorescence responses. We demonstrated that leaf Chl content within rice canopy was higher in the lower leaves during the vegetative phase, while the pattern was reversed with the highest Chl appearing in the upper leaves during the reproductive phase. The OJIP transients of leaf presented an unusual JI-fall during the vegetative phase, but it showed the typical JI-rise during the reproductive phase. The JIP parameters of leaves at different layers displayed a distinct pattern at several critical growth stages (i.e., tillering, jointing, initial heading, and late filling stages). RE_0_/CS was found to be a potential sensitive predictor for identifying the vertical heterogeneity of leaf Chl content. These findings provide an interesting perspective for future advancements on our understanding of associations between the canopy and each leaf layer of physiological traits and improving high throughput phenotyping techniques for crop breeding.

## Data Availability Statement

The raw data supporting the conclusions of this article will be made available by the authors, without undue reservation.

## Author Contributions

JZ and HC conceived and designed the experiments. JZ and LW performed the experiments. JZ and HC analyzed the data and wrote the manuscript. CI, ZZ, LW, YG, and DS made critical comments and revisions. All authors contributed to the article and approved the submitted version.

## Conflict of Interest

The authors declare that the research was conducted in the absence of any commercial or financial relationships that could be construed as a potential conflict of interest.

## Supplementary Material

Supplementary Figure 1 A simplified schematic diagram of electron transport in photosynthesis modified from Shevela and Björn (2018). Chloroplasts contain a large number of other proteins in addition to the four major membrane protein complexes (PSII; Cyt *b_6_f*; PSI; and ATPase). Mn_4_CaO_5_, manganese-calcium- oxygen complex; Y_z_, redox-active tyrosine (Tyr Z); P680 and P700, primary electron donors of Photosystem II (PSII) and Photosystem I (PSI) of the first excited states of special reaction center Chl *a* molecules; Pheo, pheophytin, primary electron acceptor of PSII; Q_A_ and Q_B_, primary and secondary quinone electron acceptors; PQ, mobile plastoquinone molecules between PSII and Cyt *b_6_f*; Fes, Rieske iron-sulfur protein; Cyt *f*, cytochrome *f*; PC, plastocyanin, mobile copper protein between Cyt *b_6_f* and PSI; A_0_, primary electron acceptor of PSI; A_1_, pair of phylloquinone (vitamin K) molecules; F_X_, F_A_, and F_B_, bound iron-sulfur clusters of PSI; F_d_, ferredoxin; FNR, ferredoxin-NADP oxidoreductase.

Supplementary Figure 2 Radar plots of each nitrogen treatment with a series of important parameters derived from experimental fast OJIP transients during different growth stages.

Supplementary Figure 3 Comparison of radar plots with important parameters during tillering, jointing, initial heading, and late filling stages in 2018 and 2019, respectively.

Supplementary Table 1 Average chlorophyll content of each layer leaf during different growth stages. Values are means ± *SD* (*n*=4). Values within a column followed by the same letters are not significantly different (*P* ≥ 0.05, Fisher’s LSD test).
