# Supplementary figures and images for: Spatiotemporal Heterogeneity of Chlorophyll Content and Fluorescence Response Within Rice (Oryza sativa L.) Canopies Under Different Nitrogen Treatments
Source: Front Plant Sci. 2021 Mar 25;12:645977. doi: 10.3389/fpls.2021.645977 (PMC8028447; doi:10.3389/fpls.2021.645977)

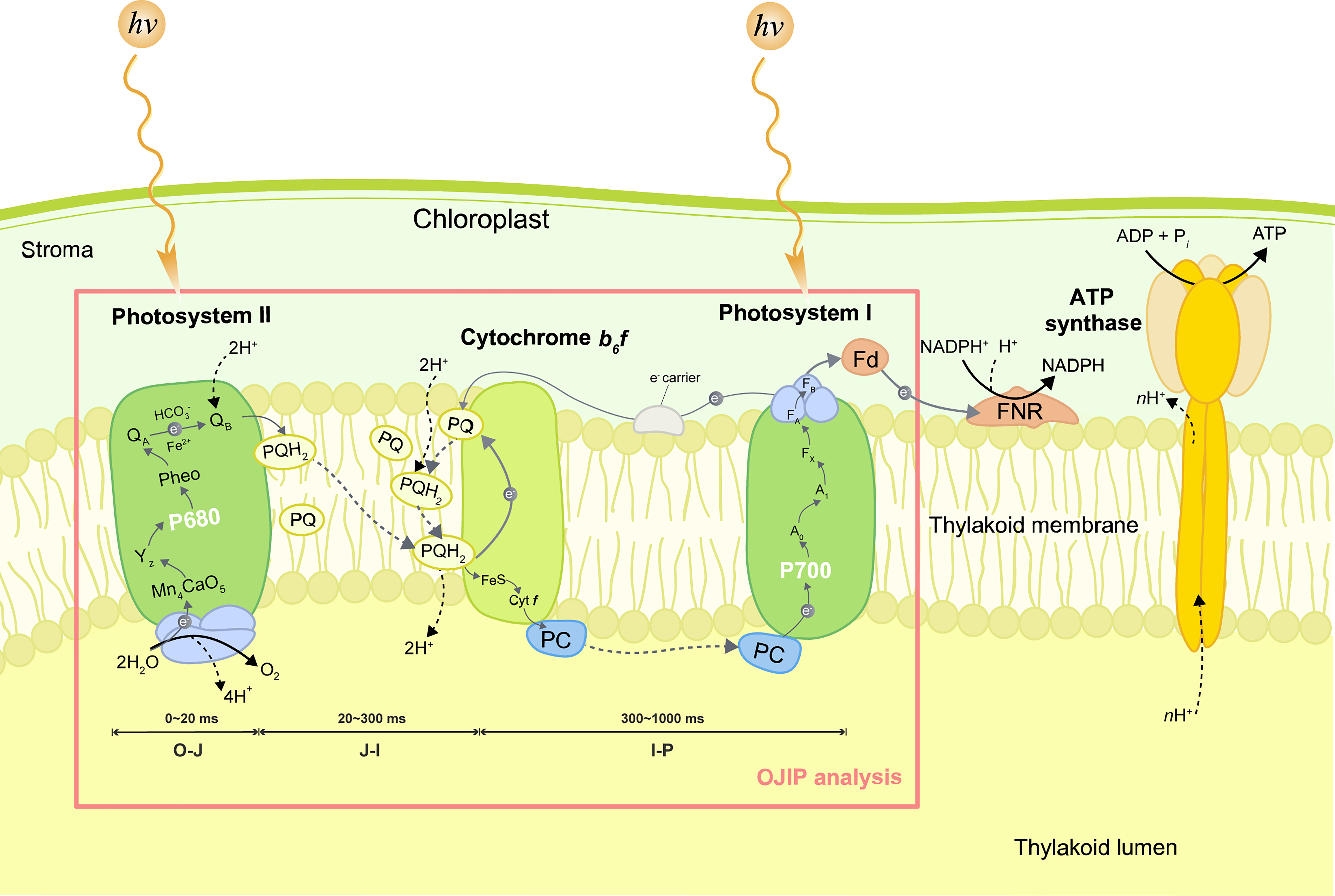

Supplement: Supplementary Figure 1 — A simplified schematic diagram of electron transport in photosynthesis modified from Shevela and Björn (2018). Chloroplasts contain a large number of other proteins in addition to the four major membrane protein complexes (PSII; Cyt b6f; PSI; and ATPase). Mn4CaO5, manganese-calcium- oxygen complex; Yz, redox-active tyrosine (Tyr Z); P680 and P700, primary electron donors of Photosystem II (PSII) and Photosystem I (PSI) of the first excited states of special reaction center Chl a molecules; Pheo, pheophytin, primary electron acceptor of PSII; QA and QB, primary and secondary quinone electron acceptors; PQ, mobile plastoquinone molecules between PSII and Cyt b6f; Fes, Rieske iron-sulfur protein; Cyt f, cytochrome f; PC, plastocyanin, mobile copper protein between Cyt b6f and PSI; A0, primary electron acceptor of PSI; A1, pair of phylloquinone (vitamin K) molecules; FX, FA, and FB, bound iron-sulfur clusters of PSI; Fd, ferredoxin; FNR, ferredoxin-NADP oxidoreductase. [file Image_1.TIF]

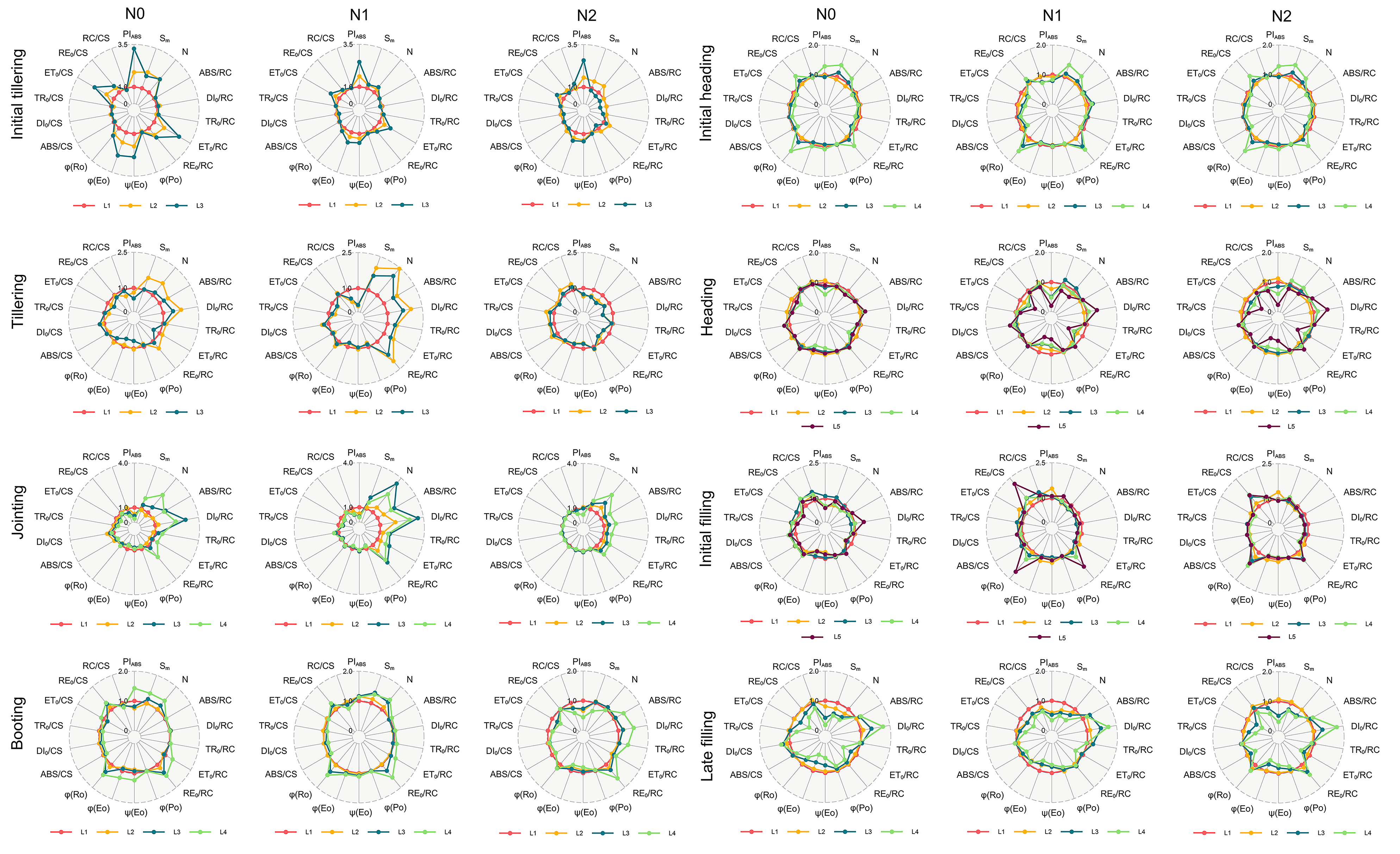

Supplement: Supplementary Figure 2 — Radar plots of each nitrogen treatment with a series of important parameters derived from experimental fast OJIP transients during different growth stages. [file Image_2.TIF]

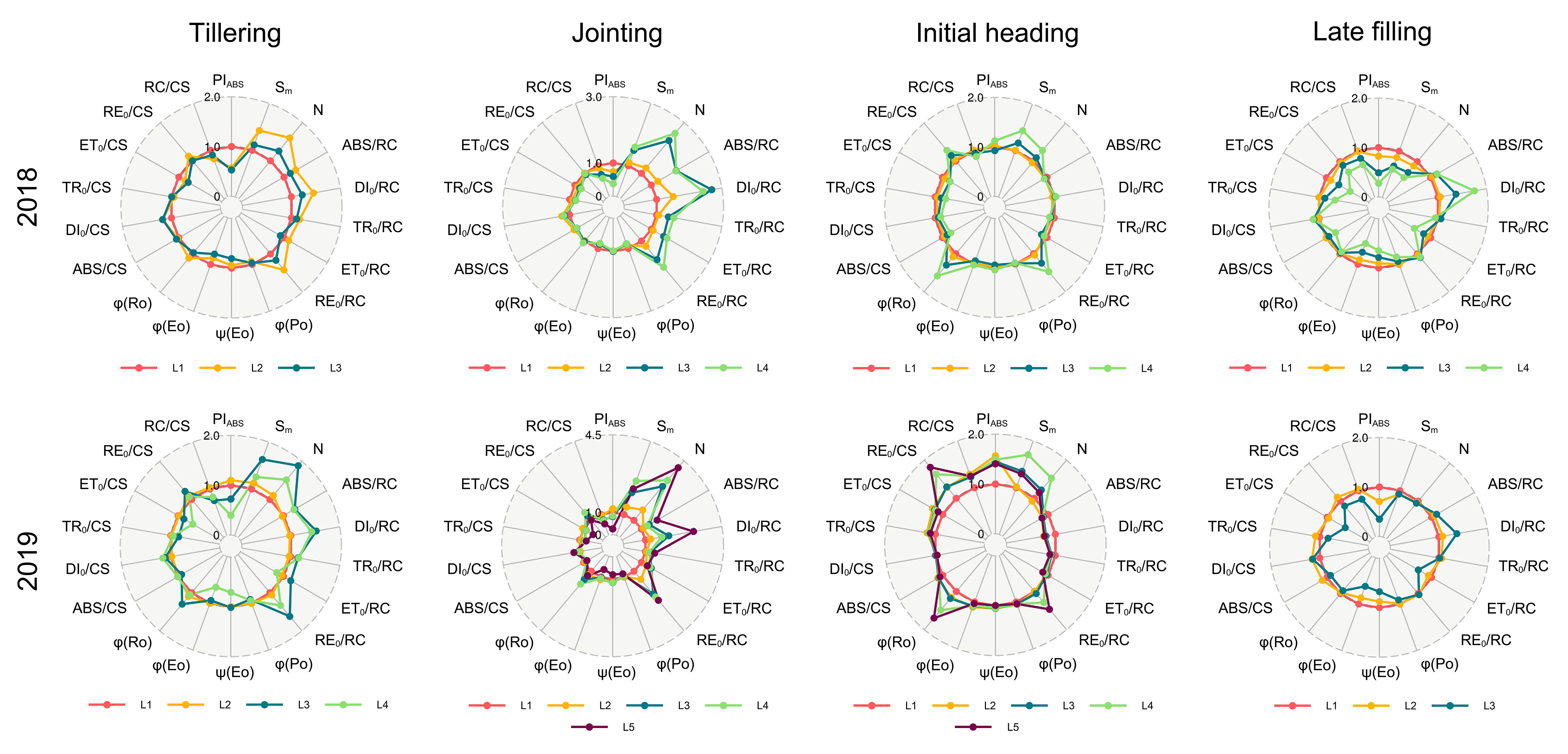

Supplement: Supplementary Figure 3 — Comparison of radar plots with important parameters during tillering, jointing, initial heading, and late filling stages in 2018 and 2019, respectively. [file Image_3.TIF]
