# Supplementary material for: Spatiotemporal Heterogeneity of Chlorophyll Content and Fluorescence Response Within Rice (Oryza sativa L.) Canopies Under Different Nitrogen Treatments
Source: Front Plant Sci. 2021 Mar 25;12:645977. doi: 10.3389/fpls.2021.645977 (PMC8028447; doi:10.3389/fpls.2021.645977)
Supplement: Supplementary Table 1 — Average chlorophyll content of each layer leaf during different growth stages. Values are means ± SD (n=4). Values within a column followed by the same letters are not significantly different (P ≥ 0.05, Fisher’s LSD test). [file Table_1.DOCX]

Supplementary Material

# Supplementary Figures and Tables

## Supplementary Figure


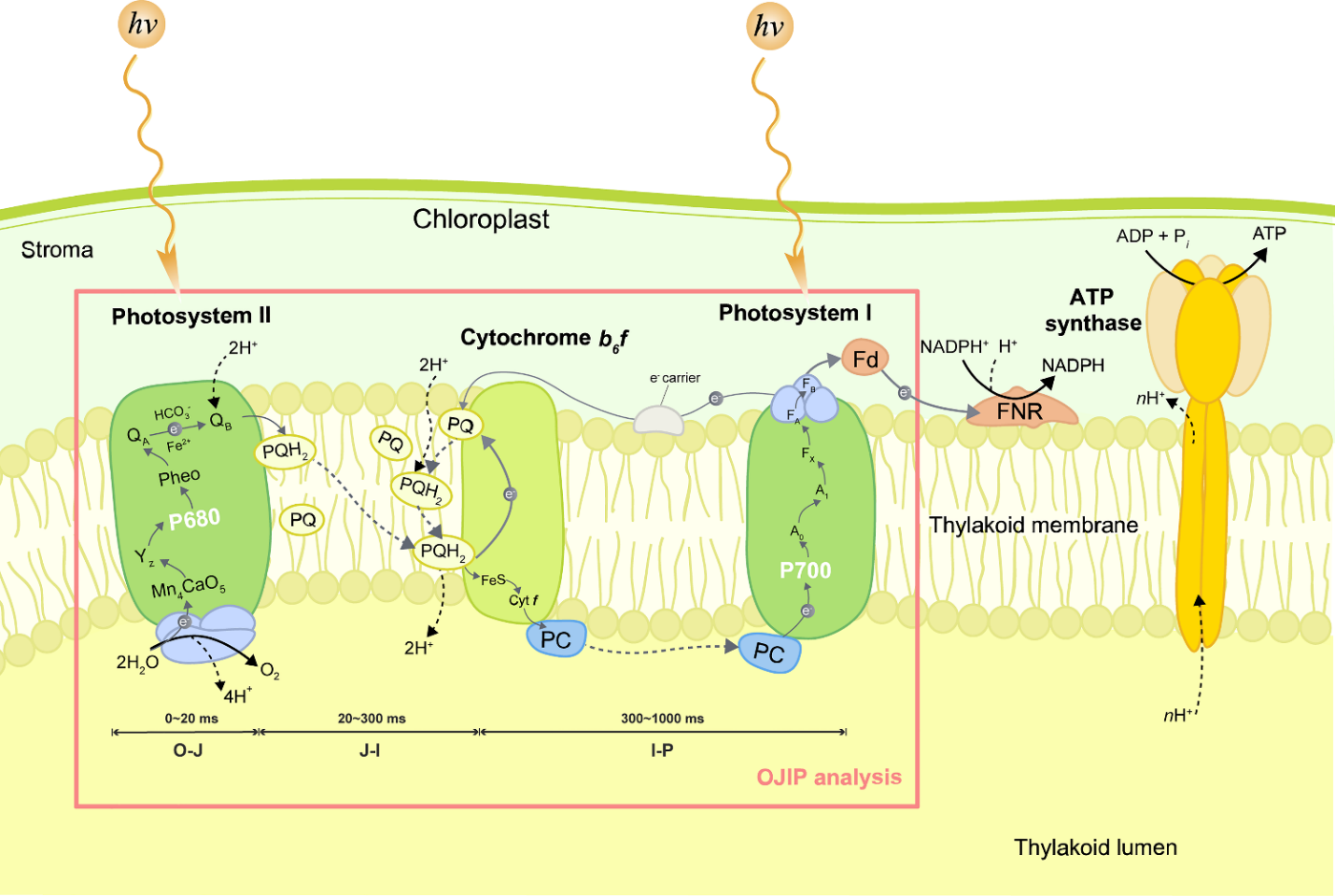


**Supplementary Figure 1.** A simplified schematic diagram of electron transport in photosynthesis modified from (Shevela and Björn, 2018). Chloroplasts contain a large number of other proteins in addition to the four major membrane protein complexes (PSII; Cyt *b_6_f*; PSI; and ATPase). Mn_4_CaO_5_, manganese-calcium-oxygen complex; Y_z_, redox-active tyrosine (Tyr Z); P680 and P700, primary electron donors of Photosystem II (PSII) and Photosystem I (PSI) of the first excited states of special reaction center Chl *a* molecules; Pheo, pheophytin, primary electron acceptor of PSII; Q_A_ and Q_B_, primary and secondary quinone electron acceptors; PQ, mobile plastoquinone molecules between PSII and Cyt *b_6_f*; Fes, Rieske iron-sulfur protein; Cyt *f*, cytochrome *f*; PC, plastocyanin, mobile copper protein between Cyt *b_6_f* and PSI; A_0_, primary electron acceptor of PSI; A_1_, pair of phylloquinone (vitamin K) molecules; F_X_, F_A_, and F_B_, bound iron-sulfur clusters of PSI; F_d_, ferredoxin; FNR, ferredoxin-NADP oxidoreductase.


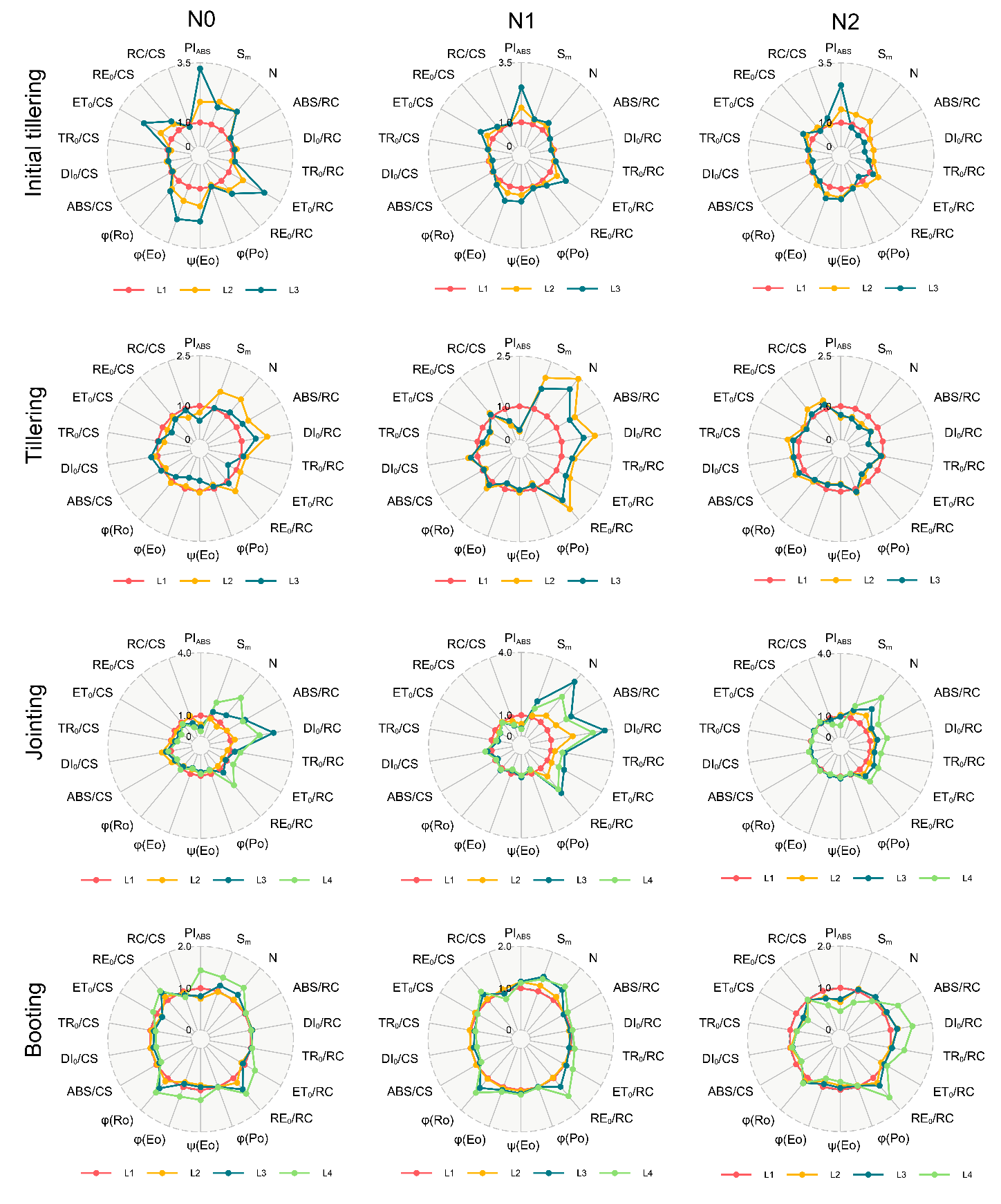


**
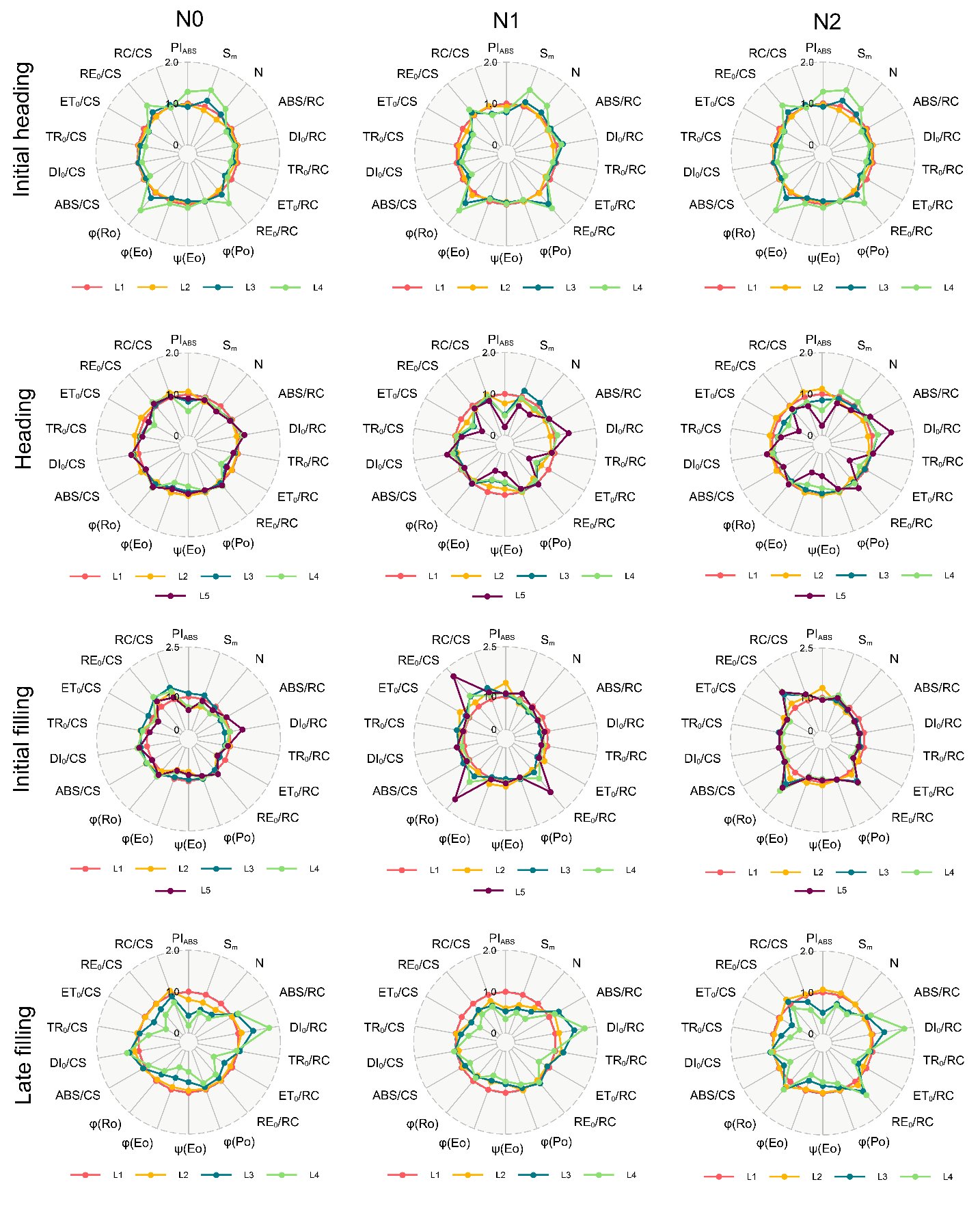
**

**Supplementary Figure 2.** Radar plots of each nitrogen treatment with a series of important parameters derived from experimental fast OJIP transients during different growth stages.


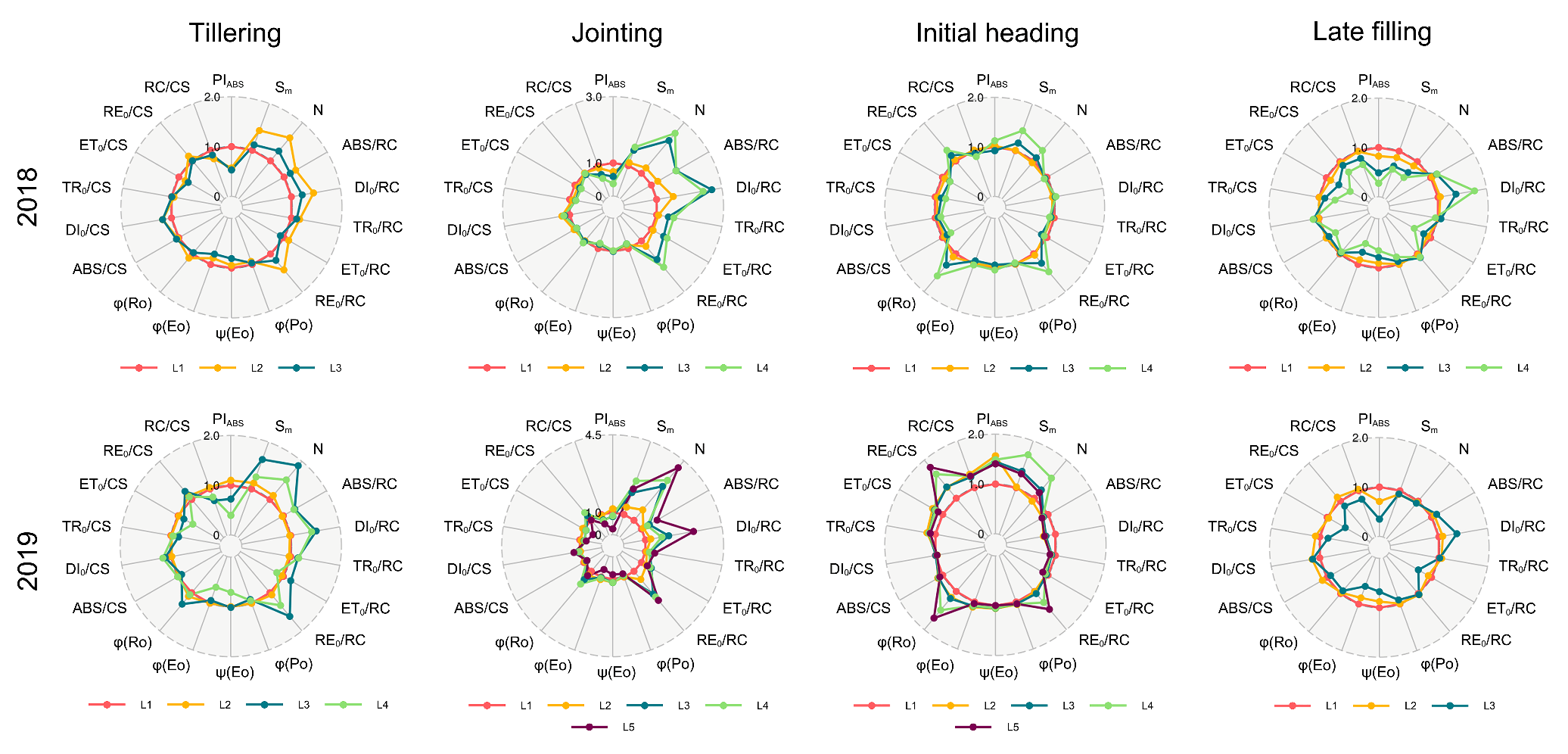


**Supplementary Figure 3.** Comparison of radar plots with important parameters during tillering, jointing, initial heading, and late filling stages in 2018 and 2019, respectively.

## Supplementary Table

| **TABLE S1 \| Average chlorophyll content of each layer leaf during different growth stages. Values are means ± SD (*n* = 4). Values within a column followed by the same letters are not significantly different (*P* ≥ 0.05, Fisher's LSD test).** | | | | | | | | | | |
| --- | --- | --- | --- | --- | --- | --- | --- | --- | --- | --- |
| Nitrogen level | Leaf position | Growth stages | | | | | | | | |
|  |  | V1 | V2 | V3 | R1 | R2 | R3 | R4 | R5 |  |
| N0 | L1 | 46.62 ± 4.76^a^ | 44.20 ± 8.43^a^ | 39.75 ± 6.44^a^ | 32.57 ± 4.09^a^ | 32.82 ± 5.00^ab^ | 21.67 ± 5.77^b^ | 12.66 ± 3.82^c^ | 8.60 ± 3.64^a^ |  |
|  | L2 | 44.25 ± 3.17^a^ | 40.63 ± 4.63^a^ | 40.60 ± 5.20^a^ | 28.19 ± 5.31^b^ | 29.82 ± 5.48^b^ | 24.53 ± 6.38^a^ | 23.02 ± 8.20^b^ | 9.50 ± 3.29^a^ |  |
|  | L3 | 32.64 ± 7.30^b^ | 36.13 ± 6.00^b^ | 39.41 ± 5.08^a^ | 27.16 ± 5.00^b^ | 32.79 ± 7.54^ab^ | 27.02 ± 6.06^ab^ | 21.90 ± 4.71^b^ | 10.57 ± 3.66^a^ |  |
|  | L4 | - | - | 29.99 ± 6.19^b^ | 27.12 ± 4.21^b^ | 34.77 ± 4.62^a^ | 29.23 ± 4.61^a^ | 25.97 ± 5.42^ab^ | 10.08 ± 3.44^a^ |  |
|  | L5 | - | - | - | - | - | 31.76 ± 3.05^a^ | 29.18 ± 5.33^a^ | - |  |
| N1 | L1 | 46.44 ± 6.08^a^ | 44.48 ± 9.44^ab^ | 43.64 ± 7.61^ab^ | 49.99 ± 4.47^a^ | 47.73 ± 4.45^a^ | 35.02 ± 7.53^a^ | 18.73 ± 9.32^c^ | 19.48 ± 7.31^a^ |  |
|  | L2 | 46.53 ± 5.23^a^ | 45.70 ± 3.93^a^ | 47.01 ± 6.60^a^ | 44.97 ± 5.01^b^ | 45.73 ± 5.49^c^ | 34.65 ± 6.34^a^ | 31.90 ± 3.94^b^ | 18.76 ± 6.01^a^ |  |
|  | L3 | 39.70 ± 7.44^b^ | 41.22 ± 8.79^b^ | 45.53 ± 6.83^a^ | 40.22 ± 4.85^c^ | 45.98 ± 4.67^c^ | 38.52 ± 4.46^a^ | 32.02 ± 4.20^b^ | 15.51 ± 5.46^a^ |  |
|  | L4 | - | - | 40.24 ± 8.97^b^ | 30.66 ± 3.09^d^ | 47.41 ± 4.88^b^ | 35.10 ± 6.58^a^ | 32.41 ± 5.27^ab^ | 15.32 ± 4.97^a^ |  |
|  | L5 | - | - | - | - | - | 39.21 ± 3.62^a^ | 36.63 ± 3.66^a^ | - |  |
| N2 | L1 | 43.64 ± 7.24^b^ | 49.43 ± 10.63^a^ | 53.13 ± 6.29^a^ | 56.87 ± 5.24^a^ | 51.39 ± 4.47^a^ | 45.37 ± 7.41^a^ | 22.34 ± 6.89^b^ | 17.99 ± 5.59^a^ |  |
|  | L2 | 48.27 ± 4.66^a^ | 48.43 ± 11.45^a^ | 54.39 ± 6.46^a^ | 50.36 ± 5.21^b^ | 43.95 ± 4.94^b^ | 46.95 ± 3.45^a^ | 36.78 ± 4.29^a^ | 16.97 ± 2.61^a^ |  |
|  | L3 | 38.54 ± 8.34^c^ | 48.53 ± 11.40^a^ | 55.12 ± 7.21^a^ | 43.27 ± 5.75^c^ | 41.07 ± 4.45^b^ | 44.36 ± 4.40^a^ | 37.03 ± 5.67^a^ | 16.27 ± 1.05^a^ |  |
|  | L4 | - | - | 41.54 ± 8.96^b^ | 30.31 ± 4.31^d^ | 41.65 ± 6.68^b^ | 40.78 ± 8.35^b^ | 37.42 ± 4.99^a^ | 17.02 ± 5.11^a^ |  |
|  | L5 | - | - | - | - | - | 43.08 ± 4.74^ab^ | 40.10 ± 4.21^a^ | - |  |
